# Adverse event profile of albumin-bound paclitaxel: a real-world pharmacovigilance analysis

**DOI:** 10.3389/fphar.2024.1448144

**Published:** 2024-10-28

**Authors:** Yuanqiong Duan, Ying Wang, Shentao Lu, Mei Zeng, Lubin Liu, Qian Dai, Rutie Yin

**Affiliations:** ^1^ Department of Obstetrics and Gynecology, West China Second University Hospital of Sichuan University, Chengdu, Sichuan, China; ^2^ Key Laboratory of Birth Defects and Related Diseases of Women and Children, Ministry of Education, Sichuan University, Chengdu, China; ^3^ Department of Obstetrics and Gynecology, Women and Children’s Hospital of Chongqing Medical University, Chongqing, China; ^4^ Department of Obstetrics and Gynecology, Chongqing Health Center for Women and Children, Chongqing, China; ^5^ Institute of Rheumatology and Immunology, Affiliated Hospital of North Sichuan Medical College, Nanchong, Sichuan, China; ^6^ Innovation Centre for Science and Technology, North Sichuan Medical College, Nanchong, Sichuan, China

**Keywords:** albumin-bound paclitaxel, adverse drug events, FDA adverse event reporting system, signal mining, pharmacovigilance analysis, real-world study

## Abstract

**Background:**

Abraxane plays a crucial role in the treatment of various types of cancer, despite the considerable attention it has garnered for its adverse drug events (ADEs). Nevertheless, there is currently a significant lack of comprehensive real-world pharmacovigilance studies on the ADEs associated with Abraxane.

**Methods:**

We conducted a retrospective analysis of ADEs associated with Abraxane using data mining from the FAERS database, analyzing data from 2005 to 2023. In a real-world setting, we quantified and visualized the signals of these ADEs using four pharmacovigilance algorithms.

**Results:**

The FAERS database identified a total of 10,230 adverse event reports associated with Abraxane. The study revealed that Abraxane-related adverse drug events involved 27 system organ classes (SOC), with the strongest signals associated with the lymphatic and hematological systems and hepatobiliary disorders. Additionally, we identified 70 significant Preferred Terms (PT) signals, which included some critical adverse events not highlighted in the product labeling, such as cystoid macular edema. Further analysis of the timing of adverse reactions showed a median onset time of 41 days. Most adverse events (AEs) occurred within the first month of using Abraxane (43.5%), although some were still possible 1 year after treatment (3.5%). Gender-specific analysis indicated that high-risk AEs differed between females (nausea, vomiting, and erythema) and males (febrile neutropenia, disseminated intravascular coagulation, and upper gastrointestinal bleeding).

**Conclusion:**

The examined results provide crucial recommendations for optimizing the administration of Abraxane, enhancing its effectiveness, and mitigating potential adverse effects. This knowledge will substantially facilitate the implementation of the substance in clinical settings.

## 1 Introduction

Taxanes, pivotal in oncology for over 50 years, have evolved from the isolation of paclitaxel to the innovative development of next-generation drugs like albumin-bound paclitaxel (nab-paclitaxel) (Mosca et al., 2021). This evolution marks a crucial advancement in cancer therapy, particularly by enhancing drug delivery and reducing side effects through novel formulations (Zierhut et al., 2019; Schiff and Horwitz, 1980). Taxanes are now widely recognized as a key chemotherapy component for several malignancies (Hellerstedt-Börjesson et al., 2018; Swain, 2011). Clinical trials have predominantly shown that taxane-based therapies excel in terms of overall response rate (ORR), progression-free survival (PFS), and overall survival (OS) (Shepherd et al., 2000; Mamounas et al., 2005; Yoneshima et al., 2021).

Despite these advancements, conventional taxanes like paclitaxel and docetaxel present challenges, including solubility issues and severe dose-limiting toxicities like peripheral sensory neuropathy and hypersensitivity reactions (Friberg et al., 2002; Shin et al., 2021; Joerger, 2012). Nab-paclitaxel, utilizing albumin-bound nanotechnology, addresses these issues effectively (Yardley, 2013; Gradishar et al., 2005). Approved by the FDA in 2005 for treatment-resistant breast cancer, nab-paclitaxel enhances solubility, reduces hydrophobicity, and improves pharmacokinetics and pharmacodynamics, resulting in increased drug accumulation in tumors and enhanced antitumor activity (Ibrahim et al., 2002). Nab-paclitaxel’s clinical trials highlighted its potential for causing significant adverse drug events (ADEs), such as neuropathy and hematological toxicities, which were manageable within trial settings but observed more severely in broader clinical use. In response, the FDA’s black box warning in 2014 underscored the risk of severe bone marrow suppression linked to nab-paclitaxel, emphasizing the need for meticulous monitoring and risk management in clinical applications (Gradishar et al., 2005; Nyman et al., 2005; Lobo et al., 2007; Higuchi et al., 2019). The thorough evaluation of these safety issues emphasized the necessity for strict monitoring and management strategies to effectively reduce these risks in clinical settings. This measure not only heightened awareness among clinicians and researchers but also highlighted the critical need for post-approval studies and continuous safety monitoring throughout the drug’s lifecycle.

The FDA’s Adverse Event Reporting System (FAERS) is a voluntary system where healthcare providers, consumers, and pharmaceutical companies are all encouraged to submit reports (Yu et al., 2021; Zhou et al., 2023). By collecting and storing data on adverse events associated with drugs and biologics used post-market, FAERS serves as a critical platform for identifying new or rare adverse events and for modifying or enhancing the understanding of known risks (Anand et al., 2019). This ultimately improves the transparency and safety of drug usage. Moreover, FAERS plays a vital role in collecting and analyzing real-world data concerning drug adverse events (AEs). This data is invaluable in filling the gaps left by pre-market research, offering insights into long-term drug safety and tolerability (Gu et al., 2023). Therefore, FAERS is indispensable for evaluating long-term safety and tolerability, offering a more comprehensive understanding that significantly augments the insights obtained from pre-market clinical research.

This study is unique in that it provides a comprehensive analysis of real-world safety data on nab-paclitaxel from the FAERS, a domain that is still relatively unexplored in existing literature. By employing advanced data mining algorithms, this research aims to provide a nuanced understanding of the risk signals associated with nab-paclitaxel, contributing significantly to its safe and rational clinical use. Our analysis not only updates the current knowledge base but also introduces new methodologies for signal detection, offering crucial insights that can influence clinical guidelines and patient management strategies.

## 2 Materials and methods

### 2.1 Data source and processing

**FIGURE 1:**
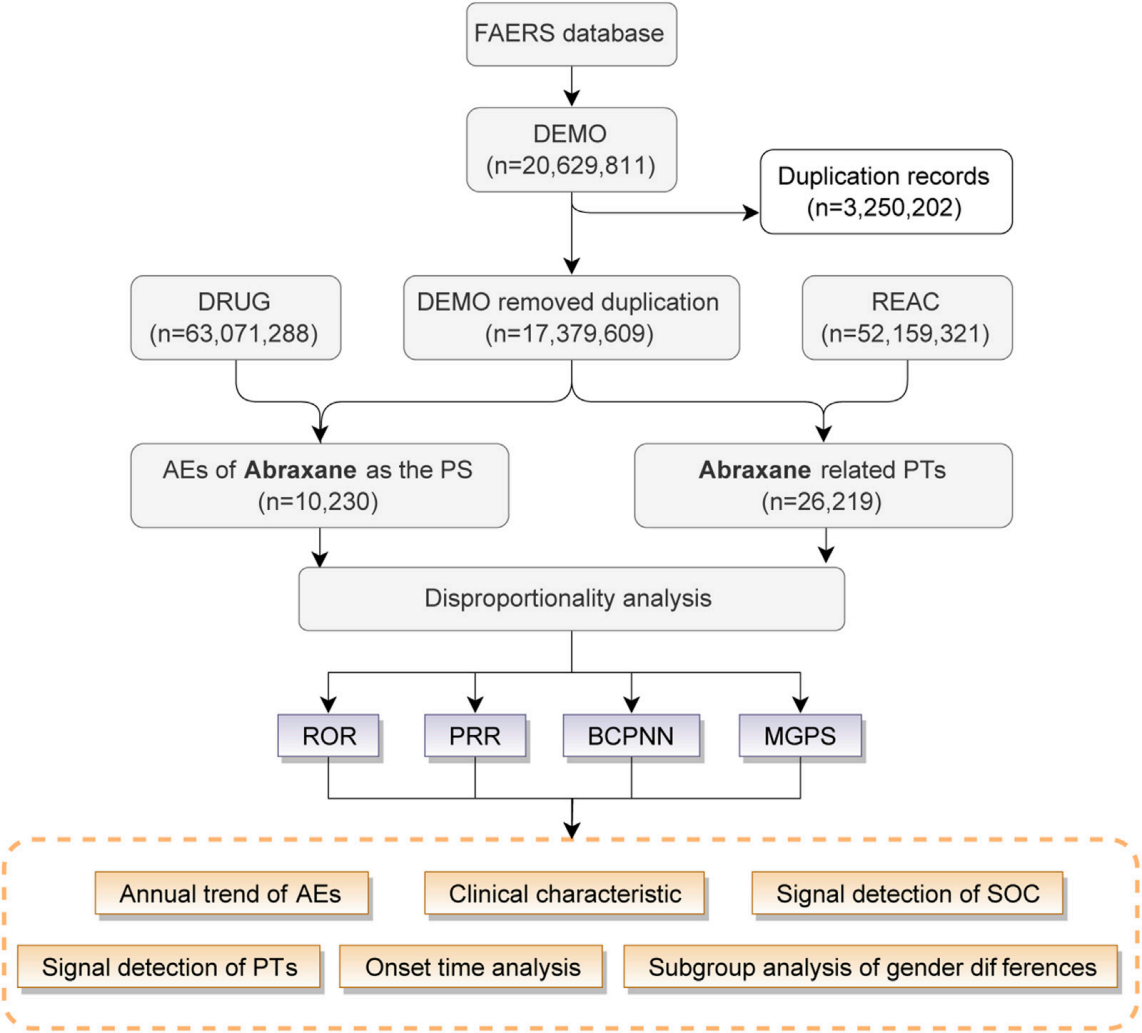
The procedure for identifying adverse drug events (ADEs) related with Abraxane form the FAERS database.

In this study, we utilized the R language to extract and analyze raw data from the FAERS database to establish an association between reported adverse drug events (ADEs) and specific drugs. Our detailed analysis concentrated on ADE reports from the FDA’s approval period in the first quarter of 2005 to the fourth quarter of 2023, identifying albumin-bound paclitaxel as the primary suspected drug. We described the drug using its generic names, “paclitaxel for injection (Albumin Bound),” “paclitaxel protein-bound particles,” “albumin-bound paclitaxel,” and its brand name, “Abraxane”. To ensure accuracy in our findings, we performed deduplication of all reports prior to conducting the statistical analysis. Figure 1 illustrates the flowchart of the study.

### 2.2 Standardization of ADE data processing

Adverse drug events (ADEs) in FAERS reports are classified using the Medical Dictionary for Regulatory Activities (MedDRA Version 26.0) Preferred Terms (PTs). Each specific AE report of Nab-paclitaxel documented at the system organ class (SOC) and PT levels was cataloged to outline the range of toxicities.

### 2.3 Data mining algorithm

In our pharmacovigilance study, we employed a disproportionality analysis to explore potential links between the drug Nab-paclitaxel and adverse events (AEs). Supplementary Table S1 contains a four-cell table that details the disproportionality methods. This study utilizes four statistical methods: the Reporting Odds Ratio (ROR), the Proportional Reporting Ratio (PRR), the Bayesian Confidence Propagation Neural Network (BCPNN), and the Multiitem Gamma Poisson Shrinker (MGPS) (Yang et al., 2023; Zhao et al., 2023). We employed the extraction rules of these algorithms to identify signals and compute scores that evaluate the relationships between drugs and AEs.

Each method applies specific criteria and equations, detailed in Supplementary Table S2, to compute scores that assess the strength of the association between the drug and reported AEs. We identify signals of AEs at the System Organ Class (SOC) level when these scores exceed predefined thresholds, indicating a strong disproportion. Moreover, our analysis identified AE signals that conformed to the criteria of all four algorithms at the preferred term (PT) level. We also noted novel AE signals, characterized as significant but not previously documented in the drug’s labeling. These findings suggest important implications for patient safety and drug usage.

In this study, we applied the Bonferroni correction to account for multiple comparisons and reduce the likelihood of Type I errors. We use the Bonferroni correction as a statistical adjustment method when testing multiple hypotheses simultaneously. It involves dividing the desired significance level (α) by the number of comparisons being made. For instance, if we set our significance level at 0.05 and conducted 10 tests, the Bonferroni-adjusted threshold for each individual test would be 0.005 (0.05/10). This adjustment ensures that the overall probability of falsely rejecting at least one null hypothesis remains at the intended significance level. We aimed to improve the reliability of our findings by implementing this correction, ensuring that any detected signals were statistically significant even after accounting for multiple testing.

### 2.4 Statistical analysis and visualization

Utilizing multivariate logistic regression, we calculated the adjusted odds ratio. The “forest plot” package facilitated the visualization of these results by producing a detailed forest diagram. We further leveraged the graphical prowess of R by using “ggplot2” and “ggpubr” for high-quality visual renditions, and employed “networkD3” packages to create informative volcano and Sankey diagrams, respectively. The volcano plot highlighted the significant variables against a backdrop of extensive testing, providing a clear link between effect sizes and their statistical significance. On the other hand, the Sankey diagram showed the clinical pathway, showing how patients responded to Abraxane treatment and how events unfolded over time. We conducted all statistical analyses and graphical illustrations in R Studio (version 4.1.2). We applied a two-tailed testing framework to adjudicate statistical significance at a *P*-value of less than 0.05.

## 3 Results

### 3.1 Descriptive analysis

**FIGURE 2:**
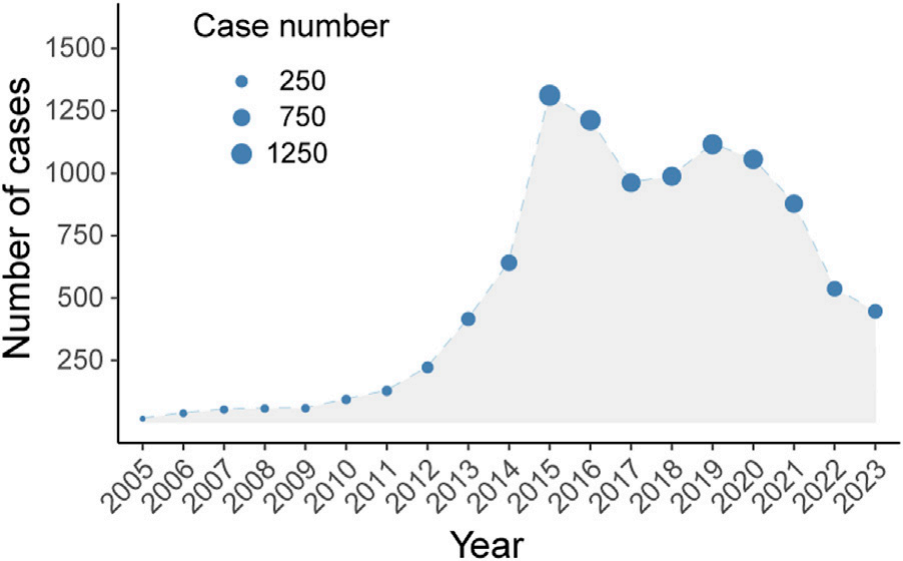
The annual trend of adverse drug event reports (ADEs) of Abraxane.

Between 2005 and 2024, the FAERS database recorded 10,230 adverse drug event (ADE) reports associated with Abraxane, as illustrated in Figure 1. The biphasic annual trend of ADE reports related to albumin-bound paclitaxel, as demonstrated in Figure 2, shows an escalation commencing in 2005, with reports cresting in 2014. A modest decline follows this initial peak, before a second rise culminates in another peak in 2018, albeit less pronounced than the first. We observe a noticeable decline in the number of reports after 2018, which continues until the fourth quarter of 2023.

**FIGURE 3:**
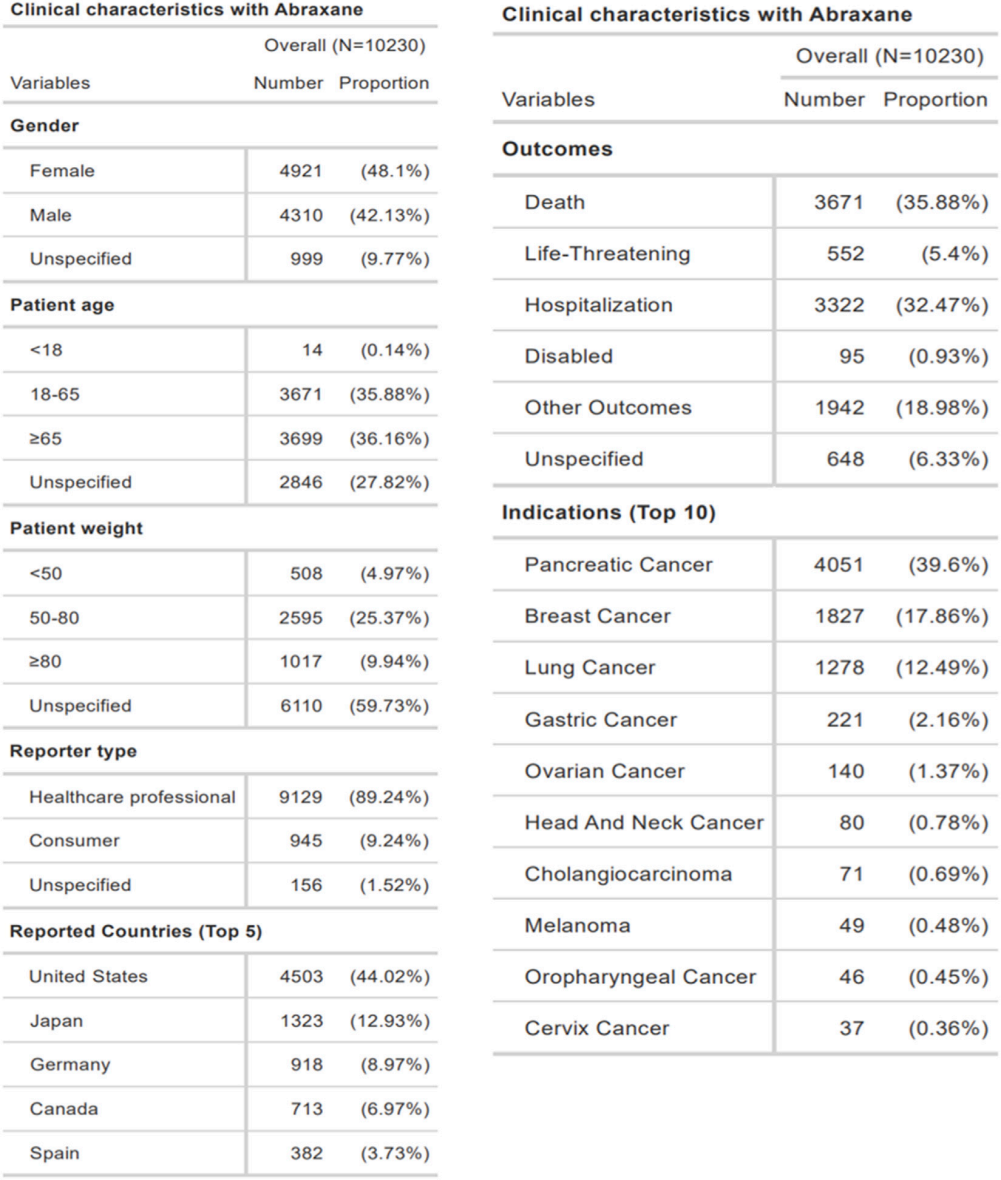
Clinical profile of ADEs associated with Abraxane in the FAERS database.

The clinical characteristics of these Nab-paclitaxel reports, indicating a slight preponderance of female (48.1%) over male (42.13%) reports, as demonstrated in Figure 3. The data revealed a concentration in adulthood: 35.88% were aged 18–65, and 36.16% were 65 years of age or older. Patient weight was reported less frequently, with 4.97% under 50 kg, 25.37% between 50 and 80 kg, and 9.94% exceeding 80 kg, while 59.73% of the reports omitted weight details. Healthcare professionals filed the bulk of the reports, accounting for 89.24% of cases, while consumers submitted 9.24%. Geographic analysis showed the United States as the predominant reporting country with 44.02% of cases, followed by Japan (12.93%), Germany (8.97%), Canada (6.97%), and Spain (3.73%). Within the reported events, death constituted the most frequent outcome at 35.88%, with hospitalization (32.47%) and other outcomes (18.98%) also being significant. 5.4% of cases reported life-threatening conditions, while 0.93% reported disability. Pancreatic cancer, at 39.6%, was the most commonly reported indication for Abraxane use, followed by breast cancer (17.86%) and lung cancer (12.49%). Reports of other malignancies, including gastric, ovarian, head and neck, cholangiocarcinoma, melanoma, oropharyngeal, and cervical cancer, were less frequent.

**FIGURE 4:**
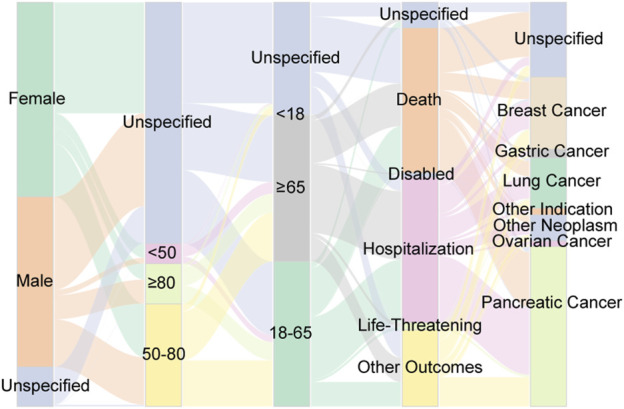
Distribution of some clinical characteristic associated with ADEs from Abraxane, including gender, age, weight, outcome and tumor type. The varying widths of the flows within the diagram quantify the volume of reports, thereby highlighting prevalent trends in the dataset.

Furthermore, we employed a Sankey diagram to illustrate the clinical characteristics linked to AEs associated with albumin-bound paclitaxel. This visual tool effectively delineates the distribution of reports, mapping out the progression from patient demographics to clinical endpoints. As depicted in Figure 4, the diagram visually encapsulates the relationships and dynamic flow between patients’ gender, age, body weight, indications for treatment, and the resulting clinical outcomes. While the Sankey diagram provides a graphical representation of the data, it is important to note that it does not imply causality or absolute frequency due to the limitations inherent in the spontaneous reporting system, such as underreporting and variability in report quality (Noguchi et al., 2021).

### 3.2 Signal detection of system organ class

**FIGURE 5:**
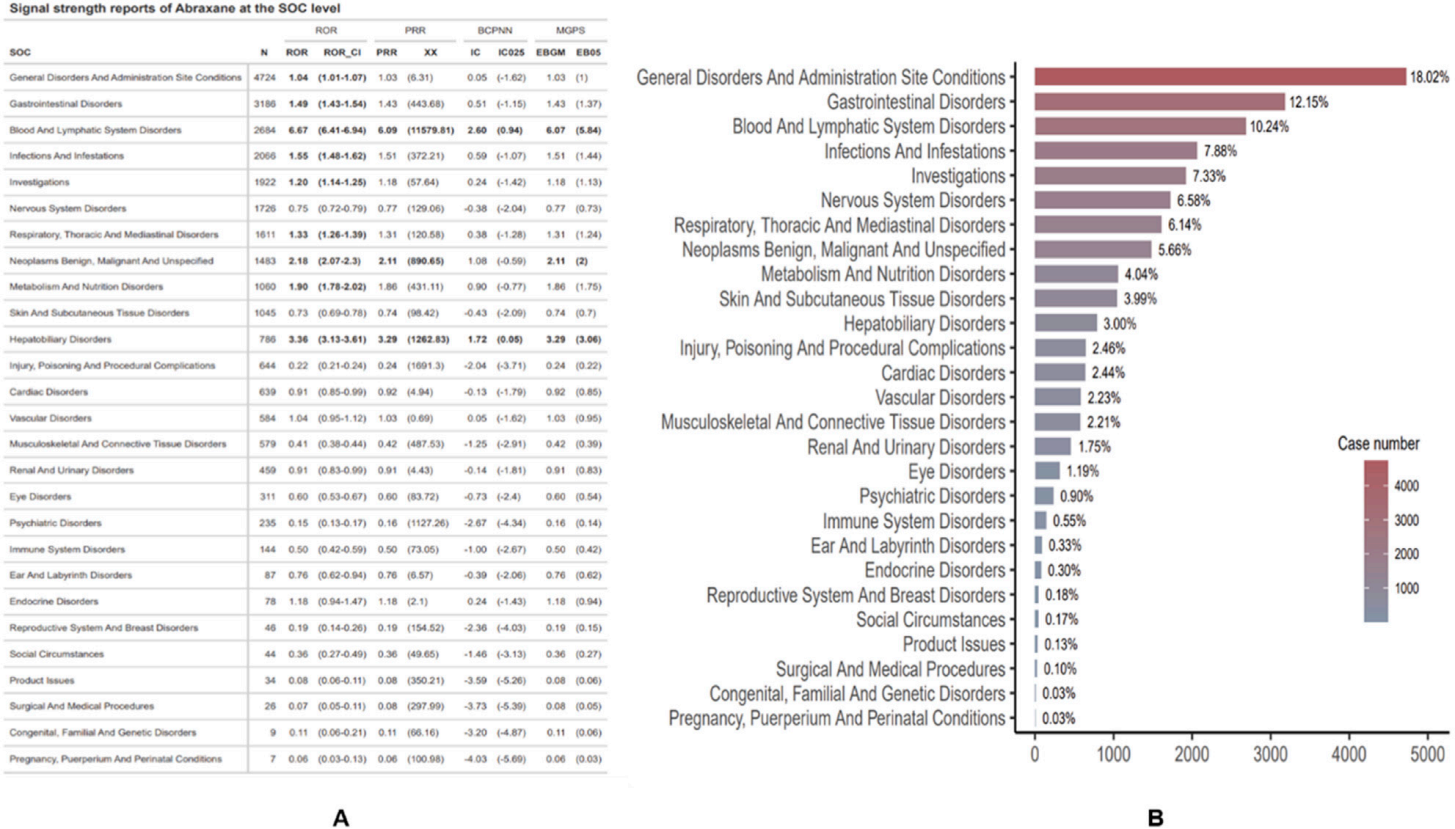
Distribution and signal strength of ADEs for Abraxane by system organ class (SOC). **(A)** Reports of Abraxane signal strength at the SOC level. **(B)** Proportional distribution of ADEs reports by SOC, detailing the percentage and total case count for each category.

Figure 5 depicts the ADE profile of albumin-bound paclitaxel, classified by System Organ Class (SOC). In relation to Nab-paclitaxel ADEs, we analyzed a total of 27 organ systems. To identify significant SOCs among these, we employed four statistical indices: the Reporting Odds Ratio (ROR), the Proportional Reporting Ratio (PRR), the Bayesian Confidence Propagation Neural Network (BCPNN), and the Multi-item Gamma Poisson Shrinker (MPGS).

Blood and lymphatic system disorders have the highest ROR (6.67 [95% CI 6.41-6.94]) among SOCs, as shown in Figure 5A. This indicates a significantly stronger signal when compared to disorders in other classes. Subsequently, hepatobiliary disorders and benign, malignant, and unspecified neoplasms occur (ROR 3.36 [95% CI 3.13–3.61]; ROR 2.18 [95% CI 2.07–2.3]). Notably, cancer patients primarily use albumin-bound paclitaxel, and their underlying conditions (such as tumors) may themselves lead to certain adverse events. For instance, the drug may not be the sole cause of hepatobiliary disorders and the occurrence of neoplasms (such as hepatobiliary system disorders and benign, malignant, and unspecified neoplasms), but rather the patient’s underlying disease. Therefore, the signals observed in the ROR may partially reflect the presence of the underlying condition rather than the true effect of the drug. With 4,724 cases, general disorders and administration site conditions were the most frequently reported SOCs. However, their relative risk (ROR) was only 1.04 (95% CI 1.01–1.07), suggesting a signal that was not as pronounced but still significant. Additionally, signal detection analysis for Abraxane at the SOC level revealed strong associations with two major categories. The blood and lymphatic system disorders reported 2,684 cases, with a ROR of 6.67 (95% CI: 6.41–6.94) and a PRR of 6.09 (XX: 11579.81). Meanwhile, 786 cases reported hepatobiliary disorders, with an ROR of 3.36 (95% CI: 3.13–3.61) and a PRR of 3.29 (XX: 1262.83). These results were backed up by more advanced pharmacovigilance algorithms, like BCPNN and MGPS. The IC and EBGM values were above their respective thresholds, which means there were strong signals. Figure 5B depicts the proportion of reports per SOC. General disorders and administration site conditions accounted for the highest proportion of reports (18.02%), followed by gastrointestinal disorders (12.15%) and blood and lymphatic system disorders (10.24%). SOCs that affect the reproductive system and breast disorders occur less frequently (0.18%), while conditions that occur during pregnancy, puerperium, and perinatal life occur sporadically (0.03%).

### 3.3 Signal detection of preferred terms and Bonferroni-adjusted *P*-value analysis

**FIGURE 6:**
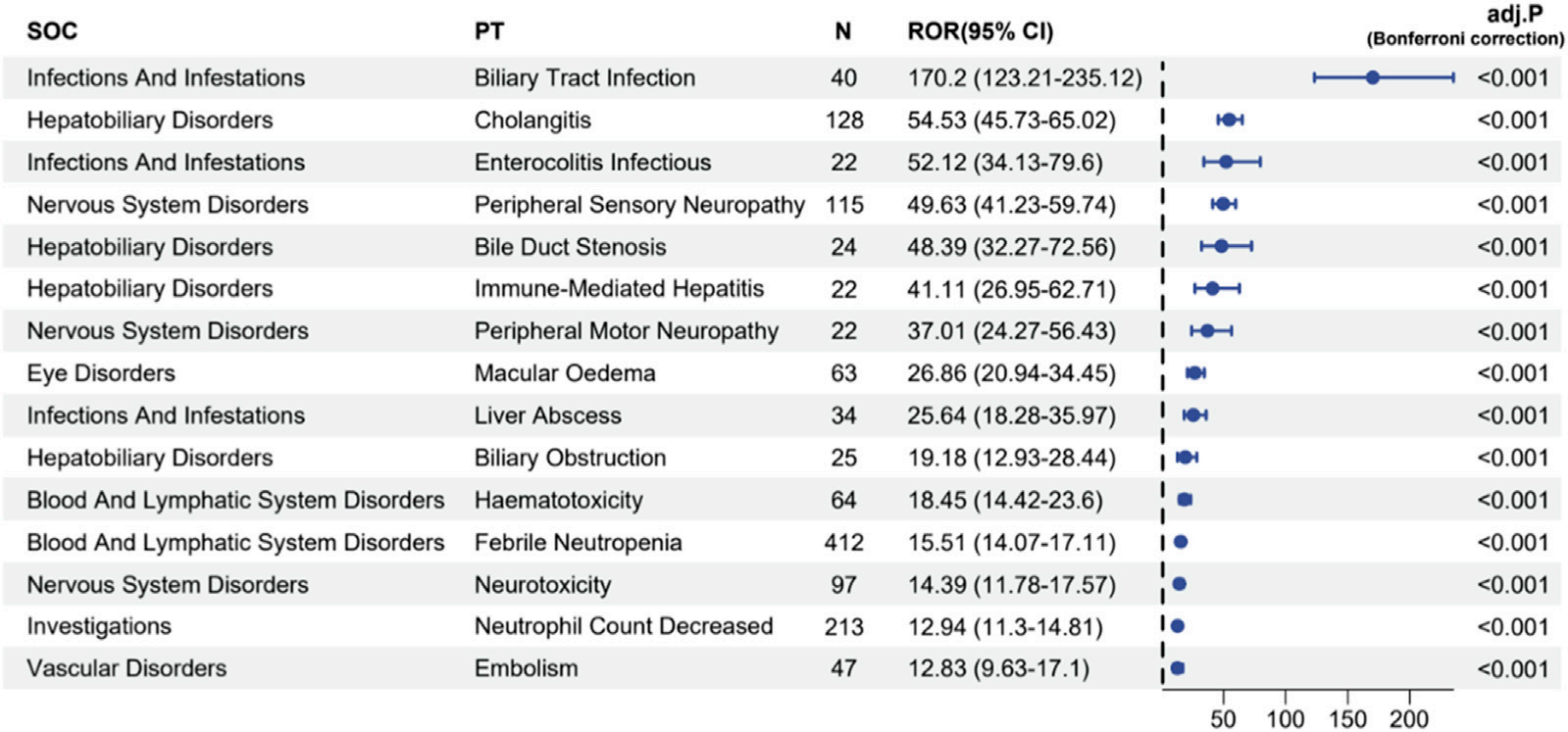
Signal strength reports of Abraxane at the preferred terms level in the FAERS database.

The four algorithms combined identified a total of 70 preferred terms (PTs) associated with Abraxane, as detailed in Supplementary Table S3. Figure 6 illustrates these PTs linked with the most pronounced signal strengths, specially highlighting the top 20% of reporting odds ratios (ROR). These ROR values signify the association’s strength between the administration of a specific drug and the incidence of reported adverse events. The analysis showed that biliary tract infection happened in 40 cases, with a high ROR of 170.2 (95% CI: 123.21–235.12), which means that there is a strong signal for this particular adverse event. Furthermore, the study documented 128 instances of cholangitis, with an ROR of 54.53 (95% CI: 45.73–65.02), and 115 instances of peripheral sensory neuropathy, with an ROR of 49.63 (95% CI: 41.23–59.74). There were 412 reports of febrile neutropenia, which is another serious side effect, with an ROR of 15.51 (95% CI: 14.07–17.11). Other adverse events with noteworthy signal strengths included a decreased neutrophil count (213 cases, ROR 12.94) and embolism (47 cases, ROR 12.83). After applying the Bonferroni correction to account for multiple testing, the associations remained highly significant (adj. *P* < 0.001), confirming the robustness of the safety signals detected for these adverse events. The spectrum of significant AEs identified through our data mining extends beyond those explicitly listed in the product label for albumin-bound paclitaxel, thus contributing to a more comprehensive understanding of the drug’s safety profile.

### 3.4 Onset time of Nab-paclitaxel associated AEs

**FIGURE 7:**
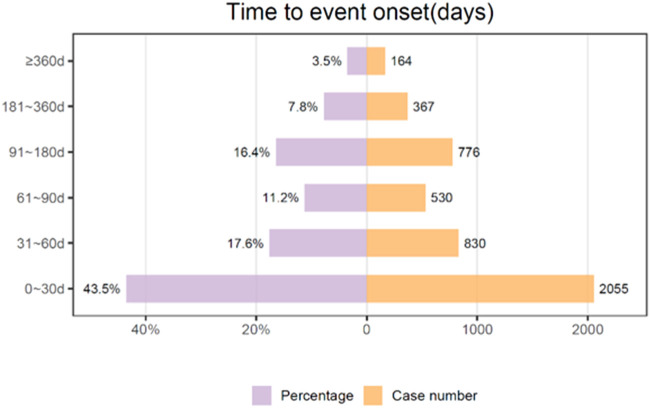
Time to onset of Abraxane associated AEs collected from the FAERS database.

We conducted a temporal analysis of adverse events associated with albumin-bound paclitaxel, and Figure 7 depicts the results. With the exclusion of reports that contained unspecified or inaccurate information concerning the timing of onset, a total of 4,722 adverse events were documented, representing 46.16% of the cases where data were accessible (4,722 out of 10,230 total reports). The median duration until the onset of the event was 41 days, and the interquartile range (IQR) was between 11 and 97 days. The data collected during the study period revealed that 43.5% of adverse events (AEs) occurred within the first 30 days following treatment initiation. The occurrence of adverse events (AEs) gradually decreased over time, with subsequent intervals of 61–90 days and 31–60 days accounting for 17.2% and 11.2% of the cases, respectively. The period spanning from 91 to 180 days exhibited a consistent decline, comprising 16.4% of the total reports. Extended adverse events (AEs) occurred with reduced frequency. Specifically, 7.8% of AEs occurred between 181 and 360 days after treatment initiation, and only a minority (3.5%) occurred after 360 days had passed.

### 3.5 Sigal of preferred terms gender difference risk

**FIGURE 8:**
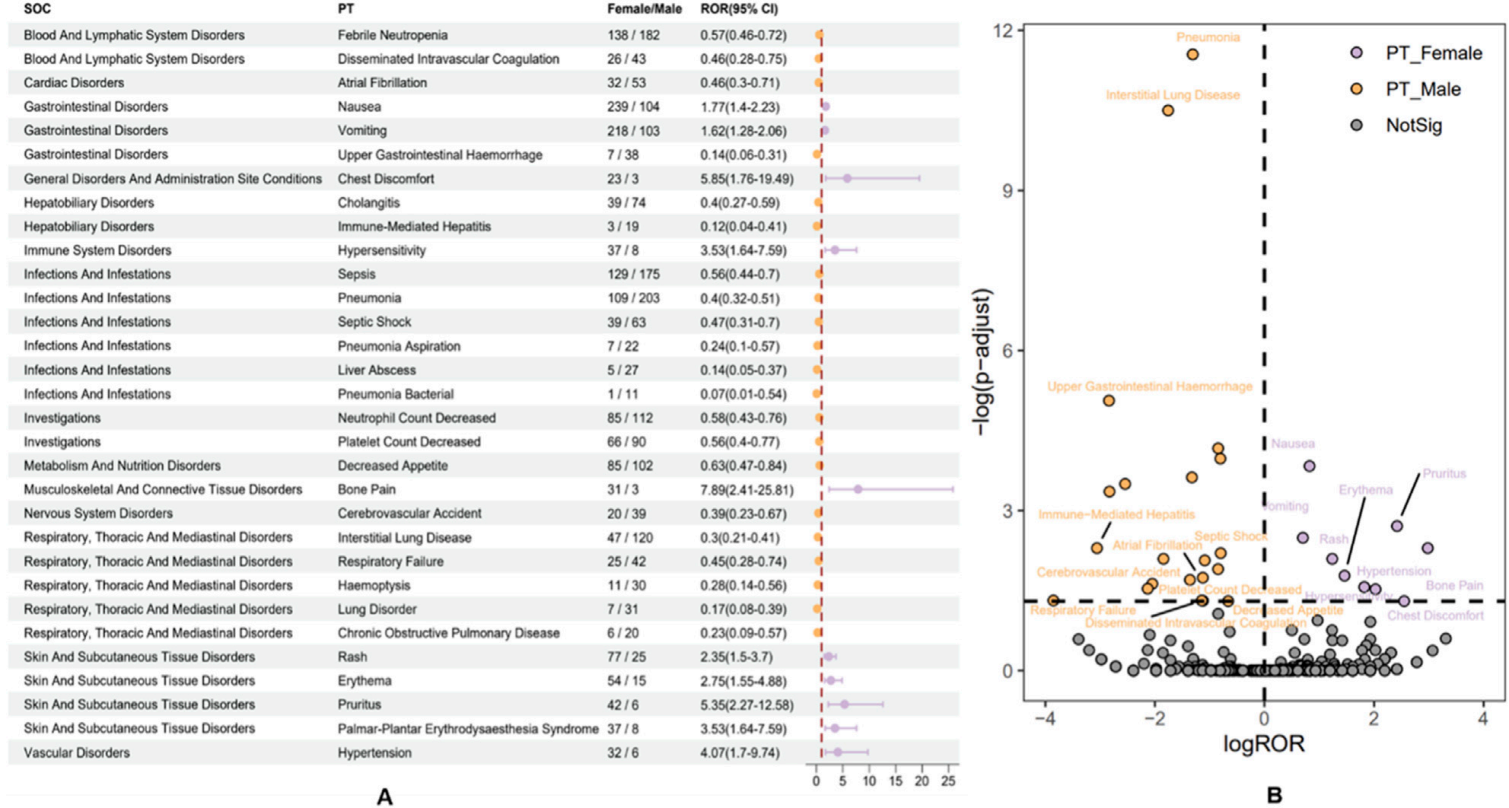
Subgroup analysis of gender differences in adverse events to Abraxane. **(A)** The various gender-specific ADEs associated with nab-paclitaxel are categorized by system organ class (SOC) and preferred term (PT), each accompanied by the corresponding ROR and 95% CI. **(B)** A volcano plot illustrates the distribution of gender differences in ADEs, highlighting significant signals with orange dots for males and purple dots for females.

After examining the FAERS database for adverse event signal strengths related to the use of albumin-bound paclitaxel, our analysis revealed gender-specific differences at the preferred term (PT) level (Figure 8A). In females, there is a notably higher incidence of adverse events such as nausea, vomiting, chest discomfort, bone pain, rash, erythema, and hypertension. Substantial reporting odds ratios (ROR) underscore these events, with conditions like bone pain exhibiting an ROR of 7.89 (95% CI: 2.41–25.81), indicating a markedly increased reporting frequency when compared to males. On the other hand, the study shows that males have stronger signals for bad events like febrile neutropenia, disseminated intravascular coagulation, upper gastrointestinal bleeding, and interstitial lung disease. Specifically, cholangitis and immune-mediated hepatitis have lower ROR values in females (0.40 and 0.12, respectively), suggesting a more pronounced risk profile in the male population.

The volcano plot (Figure 8B) distinctively visualizes the adverse drug events associated with albumin-bound paclitaxel that are specific to male and female patients. This makes it easy to find differences that are statistically significant between the two groups of patients. This graph plots the negative logarithm of the *p*-value against the log reporting odds ratio (log ROR), which pinpoints notable gender-specific signals. Events above the threshold line indicate a significant disparity in ADR reporting between genders. Specifically, orange dots represent potential ADE signals in males, while purple dots indicate those in females. Noteworthy ADEs, such as “pneumonia” and “interstitial lung disease” in males, stand out with high negative log10 *p*-values and substantial log ROR values, reflecting significant gender-based differences in their reporting. The visualization highlights the critical need to scrutinize ADEs along gender lines, as it is essential for discerning the varying risk profiles presented by pharmacotherapy across different patient groups. The emergent patterns underscore the imperative of considering gender-based variances in the safety and surveillance of albumin-bound paclitaxel, as these differences could affect clinical decision-making and patient care.

## 4 Discussion

Pharmacovigilance analysis of adverse drug reactions is of the utmost importance in order to optimize and update information regarding drug usage. It allows us to effectively address emerging risks and intricate scenarios that may arise in our ever-changing clinical practice. As clinical practice evolves, continuous monitoring is critical for addressing emergent risks and complexities in clinical practice. This endeavor is crucial to detecting potential risks and adverse effects that may emerge when administering the medication to a wider and more heterogeneous group of patients in the future. It is imperative to recognize that the conditions of clinical trials may not consistently mirror the intricacies of the real world, such as a heterogeneous patient population and a multitude of comorbidities (Bielefeldt, 2017). This underscores the importance of continuous safety surveillance and data collection in order to gain a more comprehensive understanding of the drug’s efficacy and potential hazards in a real-world context. Changes in clinical use, reporting protocols, or pharmacovigilance approaches may lead to the observed dual-peak pattern in adverse event reporting for Abraxane (Figure 2). This means that there needs to be a thorough investigation to find the main causes of these changes, as well as what effects they might have on patients’ safety and the drug’s effectiveness. This study employs the FAERS database to provide the most comprehensive pharmacovigilance review of all adverse events associated with albumin-bound paclitaxel to date. By conducting an exhaustive and methodical examination of worldwide reports concerning the medication’s detrimental impacts, this resource empowers medical practitioners to enhance clinical protocols and refine judgments. This improves patient outcomes and strengthens public trust in the drug’s long-term safety.

In the current study, we extracted 10,230 ADE reports related to albumin-bound paclitaxel from the FAERS database and analyzed their clinical characteristics (Figures 3, 4). Although some reports failed to specify gender, the data indicated a greater frequency of adverse reactions in female patients compared to their male counterparts. This observation may reflect the drug’s primary application in treating cancers prevalent in females, such as breast, ovarian, and cervical cancers (Yardley, 2013; Cortes et al., 2020; Sibaud et al., 2016). The age distribution of the reports, primarily adults aged 18-65 and elder over 65, aligns with the drug’s target demographic. Notably, healthcare personnel submitted the majority of ADE reports, which lends credence to the data’s reliability. However, the predominance of reports from Western countries introduces a potential geographic bias, underrepresenting data from Asian regions. Approximately 70% of the reports noted severe adverse drug events, such as death or prolonged hospital admissions, indicating that underlying comorbidities may influence these outcomes. These findings underscore the necessity for diligent supervision and thorough risk-benefit evaluations during treatment with albumin-bound paclitaxel, particularly in individuals with multiple comorbidities. It is crucial to approach the interpretation of these data with an understanding of the inherent limitations of the FAERS database, including incomplete data capture and potential reporting biases (Noguchi et al., 2021; Noguchi and Yoshimura, 2024). Our analysis highlights the need for ongoing monitoring and further research to better understand the correlation between the medication and severe ADEs, to mitigate risks and ensure patient safety.

In our investigation of adverse events (AEs) associated with albumin-bound paclitaxel, we examined signals across 27 system organ classes (SOCs) using four pharmacovigilance algorithms—ROR, PRR, BCPNN, and MGPS—as illustrated in Figure 5. The algorithms consistently identified robust signals for hematologic and hepatobiliary disorders, indicating a significant association with increased risk when using this chemotherapy agent. These findings align with the data reported in the package insert and highlight the necessity for vigilant monitoring of these particular adverse effects. Specifically, the most frequently reported adverse events (AEs) were general disorders and administration site conditions, which accounted for 18.02% of all AEs and primarily manifested as fever, fatigue, pain, and swelling at the injection site. These reactions underline the importance of meticulous administration and storage of the medication. Gastrointestinal reactions posed significant challenges, affecting 12.15% of patients with symptoms like nausea, vomiting, diarrhea, and mucositis, severely impacting their quality of life. Adverse events in the blood and lymphatic systems were also notably prevalent, constituting 10.24% of the reports. We observed serious conditions such as anemia, bone marrow suppression, leukopenia, and thrombocytopenia, which could increase infection risks and bleeding tendencies, sometimes requiring treatment adjustments. Other notable adverse effects involved infection and infestations (7.88%), investigations (7.33%), and nervous system disorders (6.58%), reflecting the drug’s extensive impact on multiple organ systems. This comprehensive profile necessitates a multidisciplinary approach to patient care, emphasizing the need for continuous assessment and management of these risks to ensure the safety and effectiveness of therapy. Overall, the consistency of strong signals in the blood and lymphatic system, along with hepatobiliary and neoplastic disorders, calls for enhanced scrutiny and further research to better understand the underlying mechanisms and optimize patient management strategies.

This study covers most of the ADE signals and SOC categories for albumin-bound paclitaxel, which is in line with the common side effects listed on the product label (Supplementary Table S3; Figure 6). Recently, the hematological and neurological toxicities caused by this drug have garnered attention. Studies show that albumin-bound paclitaxel mainly suppresses bone marrow. This usually happens 8–10 days after chemotherapy, which means that treatment may have to be delayed or stopped, which can affect how well it works and how long the patient lives (Higuchi et al., 2017). Other studies have demonstrated the reversibility of damage to immature hematopoietic cells and the potential mitigation of bone marrow suppression by granulocyte colony-stimulating agents (Nieuweboer et al., 2015), despite the recommendation against their routine use. Therefore, we still need effective strategies to control the incidence of neutropenia and leukopenia. Notably, this study demonstrated a significant association between albumin-bound paclitaxel and peripheral neuropathy, with a reporting odds ratio (ROR) for peripheral sensory neuropathy as high as 37.01 (95% CI: 24.271–56.43). These findings align with the adverse event data observed in clinical trials. Based on a study of 4,613 patients, this drug greatly raises the risk of grade 3 sensory neuropathy in people with non-small cell lung cancer. When used with gemcitabine for pancreatic cancer treatment, the rate of neuropathy was higher (17% vs. 1%) compared to when used alone (Tan et al., 2019). The specific mechanisms behind the neurotoxicity induced by albumin-bound paclitaxel are still unclear, but they may relate to the dose limitations of taxane drugs and the long-term adverse effects causing abnormal microtubule accumulations in axons and Schwann cells (Bae et al., 2021; Hu et al., 2019). These accumulations can interfere with normal neuronal functions, leading to pain, numbness, and other neurologically related symptoms. Currently, there are no effective preventative medications, and symptomatic treatments such as mecobalamin and the use of ice gloves and booties can somewhat alleviate these symptoms during chemotherapy. Recent studies have shown that elevated levels of interleukin-20 (IL-20) in patients during chemotherapy are closely associated with the risk of peripheral neurotoxicity (Chen et al., 2020). Inhibiting the activity of IL-20 can prevent sensory neurotoxicity without affecting the therapeutic effects of the drugs, offering new directions for future treatments. However, translating these findings into clinical practice requires further clinical trials to provide evidence-based support. These studies and their outcomes will provide more effective methods for managing neurotoxicity and improving patient quality of life.

The results of this study differ somewhat from the information on the product label. For instance, our study frequently reported macular edema, or cystoid macular edema, which is considered rare under the eye disorders category. In order to increase confidence, we further corrected the correlation statistically. When performing multiple pairwise tests on a single set of data, the Bonferroni correction, a conservative method, reduces the chances of obtaining false-positive results (Type I errors). In the context of this table, cystoid macular edema has an adjusted *p*-value of <0.001, indicating that after adjusting for multiple comparisons, the signals associated with the AE are still statistically significant. Moreover, there have been sporadic reports of cystoid macular edema associated with albumin-bound paclitaxel in recent years (Yamane et al., 2023; Ye et al., 2021). This also corroborates the reliability of our research findings to a certain extent. Consequently, regular ophthalmologic exams are necessary during the clinical use of the drug, especially if changes in visual acuity or visual field occur, with optical coherence tomography needed for early detection and timely intervention. Furthermore, this study revealed high ROR values for cholangitis, bile duct stenosis, and pseudocirrhosis under the hepatobiliary disorders category, small intestine colonitis infections under the infections and infestations category, and duodenal obstruction under gastrointestinal disorders, indicating a higher risk of these ADEs and highlighting the importance of monitoring the hepatobiliary and gastrointestinal systems. Given the significant risk associated with these ADEs, future research should focus more on understanding their mechanisms and risk factors, as well as developing appropriate management strategies.

The temporal distribution of adverse events (AEs) associated with albumin-bound paclitaxel, as depicted in Figure 7, offers significant insights into the drug’s safety profile. Notably, the highest frequency of AEs occurs within the first 30 days of treatment initiation, accounting for 43.5% of the cases. This initial period may be critical for patient monitoring and could reflect the drug’s immediate pharmacological impacts on the body. The reduction in AE occurrence over time, with 17.6% of cases reported between 31 and 60 days and 11.2% between 61 and 90 days, suggests a diminishing acute toxic effect or the possible emergence of tolerance to the drug. However, the relatively sustained percentage of AEs occurring in the 91- to 180-day range (16.4%) signals the need for ongoing vigilance beyond the acute treatment phase. This might be indicative of cumulative toxicity or delayed effects of the medication that become evident only after prolonged exposure. The fact that 7.8% of AEs were reported between 181 and 360 days and a minority of 3.5% after 1 year could be associated with long-term treatment effects or late-onset toxicity, which are often overlooked in early post-marketing surveillance. It is also possible that these extended AEs are due to persistent use of the drug in a chronic setting or indicate late-emerging sequelae from earlier treatment periods. The data underscores the necessity for a comprehensive pharmacovigilance approach that extends well beyond the initial treatment window and calls for sustained patient follow-up. Additionally, the significant drop in AEs after the first month suggests that initial dosing and patient adjustment to treatment are critical factors in managing adverse outcomes. However, the non-negligible incidence of AEs after the acute phase suggests that we should not underestimate long-term side effects. Clinicians should remain aware of the potential for both acute and delayed AEs with albumin-bound paclitaxel. This study’s temporal analysis reinforces the importance of educating patients about the possibility of late-onset AEs and the need for regular monitoring, even after the initial period of highest risk has passed. These results can also help make albumin-bound paclitaxel therapy safer by guiding risk reduction strategies like changing the dose and implementing supportive care protocols.

Our analysis of the FAERS database to investigate the gender differences in adverse event (AE) reporting for albumin-bound paclitaxel has elucidated distinct patterns in drug tolerance and safety profiles between males and females. The gender-specific differences at the preferred term (PT) level, as depicted in Figure 8A, indicate that females have a significantly higher incidence of AEs such as nausea, vomiting, chest discomfort, bone pain, rash, erythema, and hypertension. Notably, bone pain showed a remarkably high reporting odds ratio (ROR) of 7.89 (95% CI: 2.41–25.81) in females. These findings suggest that gender-related biological factors, differences in drug exposure, or reporting behavior may make women more susceptible to certain AEs or more likely to report these events. In contrast, males showed stronger signals for severe conditions such as febrile neutropenia, disseminated intravascular coagulation, upper gastrointestinal hemorrhage, and interstitial lung disease. On the other hand, females showed lower RORs for conditions like cholangitis and immune-mediated hepatitis, suggesting that males are more likely to experience these side effects. This divergence in AE profiles necessitates a gender-stratified approach in clinical practice and drug surveillance.

The volcano plot (Figure 8B) further elucidates these disparities, with a clear demarcation between statistically significant ADRs in male and female patients. Log ROR and negative log10 *p*-values effectively highlight the significantly more frequently reported ADRs in one gender compared to the other. For instance, the substantial log ROR values for “pneumonia” and “interstitial lung disease” in males emphasize the importance of considering gender when assessing ADR risk profiles. These findings have a multitude of implications. Clinically, they call for heightened awareness and potentially differential monitoring strategies for male and female patients undergoing treatment with albumin-bound paclitaxel. The pronounced gender-specific AEs identified in this study could influence treatment decisions, such as dosing regimens, supportive care measures, and even the choice of therapeutic agents. Furthermore, the results reinforce the need for gender-specific analysis in pharmacovigilance studies to accurately capture and address the drug safety concerns for each patient demographic. These findings raise questions about the underlying mechanisms that contribute to gender-specific AEs from a research perspective. Further investigation into pharmacokinetics and pharmacodynamics, hormone-drug interactions, genetic factors, and even psychosocial elements could provide a deeper understanding of the observed differences. This knowledge could pave the way for personalized medicine approaches that tailor treatments to the unique risk profiles of male and female patients, ultimately improving patient care and therapeutic outcomes. It is worth noting that the previous study employed only two algorithms for the pharmacovigilance analysis of albumin-bound paclitaxel, and the data cutoff for adverse events was in 2021 (Wang and Liu, 2023). In our current study, we further analyzed the adverse events of albumin-bound paclitaxel by utilizing four algorithms, along with the Bonferroni correction, to ensure more robust signal detection and interpretation of the results. This provides a more comprehensive analysis and enhances the reliability of our findings compared to the prior study.

We used the FAERS database, an observational and spontaneous reporting system, to gather data on drug-related adverse AEs in real-world clinical settings. Given the observational nature of this dataset, disproportionality analysis methods, such as the reporting odds ratio (ROR), do not aim to establish direct causal relationships between a drug and AEs. Instead, these methods compare the frequency of reported AEs for a specific drug with the overall frequency in the entire database to identify potential associations or signals. Using the FAERS database as an internal control system, we consider the background population (all other drugs) as a comparative baseline. This approach allows us to detect disproportionality, indicating that albumin-bound paclitaxel may be associated with a higher reporting rate for certain AEs. However, the presence of a signal indicates an association rather than direct causality. Future research should focus on conducting more in-depth sensitivity analyses that compare the frequency of AEs linked to Abraxane with those of other anticancer drugs, particularly other taxanes such as docetaxel and paclitaxel. Additionally, restricting the analysis to patients with specific tumor indications would further minimize indication bias.

The FAERS database, being a spontaneous reporting system, inherently suffers from issues like underreporting and missing information, which can distort the true frequency and severity of adverse drug events (ADEs). As highlighted in research Noguchi et al. (2021), these systems provide critical safety signals but require cautious interpretation due to their non-systematic data collection processes. The variability introduced by reports from different sources—patients, healthcare providers, and pharmaceutical companies—can lead to reporting bias, affecting the reliability of the data and the statistical signals detected. Although this study utilized advanced signal detection algorithms like ROR, PRR, BCPNN, and MGPS to enhance the robustness of our findings, these methods primarily detect signals rather than establish causality. As noted in study Noguchi and Yoshimura (2024), applying these algorithms necessitates careful consideration of data discrepancies and potential confounders. The identification of ADE signals indicates statistical associations, not causality, pointing to the need for further clinical research to validate these findings. Future research should integrate broader datasets, such as electronic health records or longitudinal studies, to provide a more detailed assessment of drug safety. Despite its limitations, this study contributes new insights into the safety of albumin-bound paclitaxel, paving the way for further investigations and emphasizing the importance of continuous safety monitoring.

## 5 Conclusion

This study utilized the FAERS database to conduct an extensive investigation and analysis of the real-world adverse reaction signals associated with albumin-bound paclitaxel, primarily aligned with the information on the drug’s label. Clinicians should be cautious not only about typical adverse events such as bone marrow suppression and neurotoxicity, but also about ocular symptoms during clinical treatment. When deemed appropriate, employ pharmacological monitoring to facilitate the judicious utilization of medications in the therapeutic environment.

## Data Availability

The datasets presented in this study can be found in online repositories. The names of the repository/repositories and accession number(s) can be found below: https://www.fda.gov/.
