# Supplementary material for: Adverse event profile of albumin-bound paclitaxel: a real-world pharmacovigilance analysis
Source: Front Pharmacol. 2024 Oct 28;15:1448144. doi: 10.3389/fphar.2024.1448144 (PMC11551030; doi:10.3389/fphar.2024.1448144)
Supplement: Supplementary file 1 [file Table1.DOCX]

**Supplementary Table 1.** Proportional imbalance method 2x2 four-grid table

| Types of drugs Number of target ADE reports Number of other ADE reports total |
| --- |
| Target drugs a b a+b  Other Medications c d b+d |
| Total a+c b+d N=a+b+c+d |
