# Supplementary material for: Adverse event profile of albumin-bound paclitaxel: a real-world pharmacovigilance analysis
Source: Front Pharmacol. 2024 Oct 28;15:1448144. doi: 10.3389/fphar.2024.1448144 (PMC11551030; doi:10.3389/fphar.2024.1448144)
Supplement: Supplementary file 2 [file Table2.DOCX]

**Supplementary Table 2.** Formulas and thresholds for ROR, PRR, BCPNN and MGPS methods

| **Algorithms** | **Equation** | **Criteria** |
| --- | --- | --- |
| ROR | ROR=ad/b/c | lower limit of 95% CI>1, N≥3 |
|  | 95%CI=e^ln (ROR)±1.96(1/a+1/b+1/c+1/d) ^0.5^ |  |
| PRR | PRR=a(c+d)/c/(a+b)  χ^2^=[(ad-bc)^2](a+b+c+d)/[(a+b)(c+d)(a+c)(b+d)] | PRR≥2, χ^2^≥4, N≥3 |
| BCPNN | IC=log_2_a(a+b+c+d) (a+c) (a+b) | IC025>0 |
|  | 95%CI= E(IC) ± 2V(IC)^0.5 |  |
| MGPS | EBGM=a(a+b+c+d)/(a+c)/(a+b)  95%CI=e^ln(EBGM)±1.96(1/a+1/b+1/c+1/d)^0.5^ | EBGM05>2 |

**Notes:** Equation: a, number of reports containing both the target drug and target adverse drug reaction; b, number of reports containing other adverse drug reaction of the target drug; c, number of reports containing the target adverse drug reaction of other drugs; d, number of reports containing other drugs and other adverse drug reactions. 95%CI, 95% confidence interval; *N*, the number of reports; χ^2^, chi-squared; IC, information component; IC025, the lower limit of 95% CI of the IC; E(IC), the IC expectations; V(IC), the variance of IC; EBGM, empirical Bayesian geometric mean; EBGM05, the lower limit of 95% CI of EBGM.
