# Supplementary material for: Adverse event profile of albumin-bound paclitaxel: a real-world pharmacovigilance analysis
Source: Front Pharmacol. 2024 Oct 28;15:1448144. doi: 10.3389/fphar.2024.1448144 (PMC11551030; doi:10.3389/fphar.2024.1448144)
Supplement: Supplementary file 3 [file Table3.DOCX]

| **Supplementary Table 3.** Signal strength reports of Abraxane at the PT level | | | | | | | | | | |  |
| --- | --- | --- | --- | --- | --- | --- | --- | --- | --- | --- | --- |
|  |  | ROR | | PRR | | BCPNN | | MGPS | | Bonferroni | |
| PTs | Cases | ROR | ROR_CI | PRR | χ^2^ | IC | IC025 | EBGM | EBGM05 | adj.P | |
| **Blood And Lymphatic System Disorders** | | | | | | | | | | |  |
| Neutropenia | 556 | 10.13 | (9.31-11.02) | 9.93 | (4454.42) | 3.31 | (1.64) | 9.89 | (9.22) | <0.001 | |
| Anaemia | 480 | 5.88 | (5.37-6.43) | 5.79 | (1901.18) | 2.53 | (0.86) | 5.77 | (5.35) | <0.001 | |
| Febrile Neutropenia | 412 | 15.51 | (14.07-17.11) | 15.29 | (5464.83) | 3.92 | (2.26) | 15.18 | (13.99) | <0.001 | |
| Thrombocytopenia | 370 | 8.00 | (7.22-8.87) | 7.90 | (2226.3) | 2.98 | (1.31) | 7.88 | (7.23) | <0.001 | |
| Leukopenia | 201 | 9.60 | (8.35-11.03) | 9.54 | (1529.54) | 3.25 | (1.58) | 9.49 | (8.45) | <0.001 | |
| Bone Marrow Failure | 101 | 10.71 | (8.8-13.03) | 10.67 | (880.98) | 3.41 | (1.74) | 10.62 | (9.01) | <0.001 | |
| Myelosuppression | 99 | 11.77 | (9.66-14.35) | 11.73 | (966.3) | 3.54 | (1.88) | 11.67 | (9.89) | <0.001 | |
| Pancytopenia | 79 | 3.42 | (2.74-4.26) | 3.41 | (134.55) | 1.77 | (0.1) | 3.41 | (2.83) | <0.001 | |
| Disseminated Intravascular Coagulation | 70 | 11.17 | (8.83-14.13) | 11.14 | (642.96) | 3.47 | (1.8) | 11.09 | (9.11) | <0.001 | |
| Haematotoxicity | 64 | 18.45 | (14.42-23.6) | 18.40 | (1043.74) | 4.19 | (2.52) | 18.24 | (14.84) | <0.001 | |
| Lymphopenia | 42 | 7.12 | (5.26-9.64) | 7.11 | (219.73) | 2.83 | (1.16) | 7.09 | (5.5) | <0.001 | |
| Thrombotic Microangiopathy | 21 | 5.56 | (3.62-8.54) | 5.56 | (78.28) | 2.47 | (0.8) | 5.55 | (3.87) | <0.001 | |
| **Cardiac Disorders** | | | | | | | | | | |  |
| Pericardial Effusion | 40 | 4.17 | (3.06-5.69) | 4.17 | (96.16) | 2.06 | (0.39) | 4.16 | (3.21) | <0.001 | |
| **Eye Disorders** | | | | | | | | | | |  |
| Macular Oedema | 63 | 26.86 | (20.94-34.45) | 26.80 | (1544.14) | 4.73 | (3.06) | 26.46 | (21.48) | <0.001 | |
| **Gastrointestinal Disorders** | | | | | | | | | | |  |
| Stomatitis | 116 | 4.62 | (3.85-5.54) | 4.60 | (326.36) | 2.20 | (0.53) | 4.59 | (3.94) | <0.001 | |
| Ascites | 102 | 8.02 | (6.6-9.74) | 7.99 | (621.66) | 2.99 | (1.33) | 7.96 | (6.77) | <0.001 | |
| Colitis | 86 | 5.69 | (4.6-7.03) | 5.67 | (330.1) | 2.50 | (0.83) | 5.66 | (4.74) | <0.001 | |
| Upper Gastrointestinal Haemorrhage | 47 | 6.31 | (4.74-8.41) | 6.30 | (209.05) | 2.65 | (0.99) | 6.29 | (4.95) | <0.001 | |
| Small Intestinal Obstruction | 41 | 7.98 | (5.87-10.85) | 7.97 | (249.05) | 2.99 | (1.32) | 7.94 | (6.14) | <0.001 | |
| Ileus | 35 | 6.89 | (4.94-9.6) | 6.88 | (175.23) | 2.78 | (1.11) | 6.86 | (5.19) | <0.001 | |
| Enterocolitis | 26 | 11.90 | (8.09-17.49) | 11.88 | (257.67) | 3.56 | (1.9) | 11.82 | (8.56) | <0.001 | |
| Oesophagitis | 26 | 5.62 | (3.82-8.26) | 5.61 | (98.35) | 2.49 | (0.82) | 5.60 | (4.06) | <0.001 | |
| **General Disorders And Administration Site Conditions** | | | | | | | | | | |  |
| Death | 1858 | 5.50 | (5.25-5.77) | 5.18 | (6341.2) | 2.37 | (0.7) | 5.17 | (4.97) | <0.001 | |
| General Physical Health Deterioration | 162 | 3.62 | (3.1-4.22) | 3.60 | (304.43) | 1.85 | (0.18) | 3.60 | (3.16) | <0.001 | |
| Mucosal Inflammation | 135 | 12.51 | (10.56-14.82) | 12.45 | (1413.31) | 3.63 | (1.96) | 12.38 | (10.74) | <0.001 | |
| Generalised Oedema | 27 | 5.27 | (3.61-7.69) | 5.27 | (93.08) | 2.39 | (0.73) | 5.25 | (3.83) | <0.001 | |
| **Hepatobiliary Disorders** | | | | | | | | | | |  |
| Cholangitis | 128 | 54.53 | (45.73-65.02) | 54.27 | (6515.32) | 5.72 | (4.06) | 52.85 | (45.61) | <0.001 | |
| Hepatic Function Abnormal | 78 | 5.14 | (4.12-6.42) | 5.13 | (258.76) | 2.36 | (0.69) | 5.12 | (4.25) | <0.001 | |
| Hepatic Failure | 60 | 4.56 | (3.54-5.88) | 4.56 | (166.23) | 2.19 | (0.52) | 4.55 | (3.68) | <0.001 | |
| Jaundice | 47 | 3.91 | (2.93-5.2) | 3.90 | (101.27) | 1.96 | (0.3) | 3.90 | (3.07) | <0.001 | |
| Hepatotoxicity | 34 | 3.74 | (2.67-5.23) | 3.73 | (67.98) | 1.90 | (0.23) | 3.73 | (2.81) | <0.001 | |
| Cholecystitis | 26 | 5.21 | (3.55-7.66) | 5.21 | (88.18) | 2.38 | (0.71) | 5.20 | (3.77) | <0.001 | |
| Biliary Obstruction | 25 | 19.18 | (12.93-28.44) | 19.16 | (426.3) | 4.25 | (2.58) | 18.99 | (13.66) | <0.001 | |
| Bile Duct Stenosis | 24 | 48.39 | (32.27-72.56) | 48.35 | (1086.49) | 5.56 | (3.89) | 47.23 | (33.65) | <0.001 | |
| Hypertransaminasaemia | 23 | 10.78 | (7.15-16.24) | 10.77 | (202.78) | 3.42 | (1.76) | 10.72 | (7.61) | <0.001 | |
| Immune-Mediated Hepatitis | 22 | 41.11 | (26.95-62.71) | 41.07 | (842.78) | 5.33 | (3.66) | 40.26 | (28.28) | <0.001 | |
| **Infections And Infestations** | | | | | | | | | | |  |
| Sepsis | 336 | 7.10 | (6.37-7.9) | 7.02 | (1730.97) | 2.81 | (1.14) | 7.00 | (6.39) | <0.001 | |
| Septic Shock | 110 | 6.22 | (5.15-7.5) | 6.20 | (478.08) | 2.63 | (0.96) | 6.18 | (5.28) | <0.001 | |
| Biliary Tract Infection | 40 | 170.20 | (123.21-235.12) | 169.95 | (6189.16) | 7.29 | (5.62) | 156.64 | (119.54) | <0.001 | |
| Device Related Infection | 36 | 4.84 | (3.49-6.72) | 4.84 | (109.36) | 2.27 | (0.61) | 4.83 | (3.67) | <0.001 | |
| Pneumocystis Jirovecii Pneumonia | 35 | 6.90 | (4.95-9.61) | 6.89 | (175.65) | 2.78 | (1.11) | 6.87 | (5.2) | <0.001 | |
| Liver Abscess | 34 | 25.64 | (18.28-35.97) | 25.61 | (793.81) | 4.66 | (2.99) | 25.29 | (19.06) | <0.001 | |
| Neutropenic Sepsis | 32 | 10.07 | (7.11-14.25) | 10.06 | (259.76) | 3.32 | (1.66) | 10.01 | (7.49) | <0.001 | |
| Enterocolitis Infectious | 22 | 52.12 | (34.13-79.6) | 52.08 | (1073.99) | 5.67 | (4) | 50.77 | (35.62) | <0.001 | |
| **Investigations** | | | | | | | | | | |  |
| Neutrophil Count Decreased | 213 | 12.94 | (11.3-14.81) | 12.84 | (2311.95) | 3.67 | (2.01) | 12.76 | (11.4) | <0.001 | |
| White Blood Cell Count Decreased | 191 | 4.13 | (3.58-4.76) | 4.11 | (449.17) | 2.04 | (0.37) | 4.10 | (3.64) | <0.001 | |
| Platelet Count Decreased | 165 | 3.62 | (3.1-4.22) | 3.60 | (310.02) | 1.85 | (0.18) | 3.60 | (3.16) | <0.001 | |
| Alanine Aminotransferase Increased | 109 | 4.04 | (3.35-4.88) | 4.03 | (247.95) | 2.01 | (0.34) | 4.02 | (3.44) | <0.001 | |
| Aspartate Aminotransferase Increased | 101 | 4.33 | (3.56-5.26) | 4.32 | (256.92) | 2.11 | (0.44) | 4.31 | (3.66) | <0.001 | |
| Blood Bilirubin Increased | 73 | 6.11 | (4.86-7.7) | 6.10 | (310.44) | 2.61 | (0.94) | 6.08 | (5.02) | <0.001 | |
| Transaminases Increased | 56 | 5.87 | (4.51-7.63) | 5.86 | (224.93) | 2.55 | (0.88) | 5.84 | (4.69) | <0.001 | |
| Blood Alkaline Phosphatase Increased | 46 | 4.08 | (3.05-5.44) | 4.07 | (106.36) | 2.02 | (0.36) | 4.06 | (3.19) | <0.001 | |
| **Metabolism And Nutrition Disorders** | | | | | | | | | | |  |
| Dehydration | 190 | 3.30 | (2.86-3.8) | 3.28 | (301.62) | 1.71 | (0.05) | 3.28 | (2.91) | <0.001 | |
| Hyponatraemia | 90 | 3.70 | (3.01-4.55) | 3.69 | (176.53) | 1.88 | (0.22) | 3.69 | (3.1) | <0.001 | |
| Hypokalaemia | 75 | 3.88 | (3.09-4.87) | 3.87 | (159.39) | 1.95 | (0.28) | 3.86 | (3.2) | <0.001 | |
| Hyperglycaemia | 65 | 4.12 | (3.23-5.26) | 4.12 | (153.06) | 2.04 | (0.37) | 4.11 | (3.35) | <0.001 | |
| Hypophagia | 48 | 4.02 | (3.02-5.33) | 4.01 | (108.29) | 2.00 | (0.34) | 4.00 | (3.16) | <0.001 | |
| Hypoalbuminaemia | 32 | 10.09 | (7.13-14.28) | 10.08 | (260.31) | 3.33 | (1.66) | 10.03 | (7.5) | <0.001 | |
| Hypocalcaemia | 30 | 3.78 | (2.64-5.4) | 3.77 | (61.03) | 1.91 | (0.25) | 3.77 | (2.79) | <0.001 | |
| Failure To Thrive | 21 | 9.08 | (5.91-13.94) | 9.07 | (150.18) | 3.18 | (1.51) | 9.04 | (6.31) | <0.001 | |
| **Nervous System Disorders** | | | | | | | | | | |  |
| Neuropathy Peripheral | 353 | 9.17 | (8.26-10.19) | 9.06 | (2524.47) | 3.17 | (1.51) | 9.03 | (8.27) | <0.001 | |
| Peripheral Sensory Neuropathy | 115 | 49.63 | (41.23-59.74) | 49.42 | (5323.72) | 5.59 | (3.93) | 48.24 | (41.31) | <0.001 | |
| Neurotoxicity | 97 | 14.39 | (11.78-17.57) | 14.34 | (1195.02) | 3.83 | (2.17) | 14.24 | (12.04) | <0.001 | |
| Polyneuropathy | 42 | 8.69 | (6.42-11.77) | 8.68 | (284.11) | 3.11 | (1.45) | 8.64 | (6.71) | <0.001 | |
| Facial Paralysis | 31 | 4.98 | (3.5-7.09) | 4.98 | (98.29) | 2.31 | (0.65) | 4.97 | (3.7) | <0.001 | |
| Peripheral Motor Neuropathy | 22 | 37.01 | (24.27-56.43) | 36.98 | (756.05) | 5.18 | (3.51) | 36.32 | (25.52) | <0.001 | |
| **Respiratory, Thoracic And Mediastinal Disorders** | | | | | | | | | | |  |
| Interstitial Lung Disease | 175 | 8.90 | (7.67-10.33) | 8.85 | (1213.85) | 3.14 | (1.47) | 8.81 | (7.78) | <0.001 | |
| Pleural Effusion | 136 | 5.16 | (4.36-6.11) | 5.14 | (452.85) | 2.36 | (0.69) | 5.13 | (4.45) | <0.001 | |
| Pneumonitis | 123 | 11.54 | (9.67-13.79) | 11.49 | (1172.24) | 3.52 | (1.85) | 11.43 | (9.85) | <0.001 | |
| Hypoxia | 49 | 3.35 | (2.53-4.43) | 3.34 | (80.39) | 1.74 | (0.07) | 3.34 | (2.64) | <0.001 | |
| Haemoptysis | 47 | 3.87 | (2.91-5.16) | 3.87 | (99.84) | 1.95 | (0.28) | 3.86 | (3.04) | <0.001 | |
| Acute Respiratory Distress Syndrome | 33 | 4.37 | (3.1-6.15) | 4.36 | (85.39) | 2.12 | (0.46) | 4.36 | (3.27) | <0.001 | |
| **Skin And Subcutaneous Tissue Disorders** | | | | | | | | | | |  |
| Palmar-Plantar Erythrodysaesthesia Syndrome | 68 | 6.84 | (5.39-8.68) | 6.82 | (336.96) | 2.77 | (1.1) | 6.80 | (5.57) | <0.001 | |
| Nail Disorder | 25 | 7.57 | (5.11-11.21) | 7.56 | (141.88) | 2.91 | (1.25) | 7.54 | (5.43) | <0.001 | |
| **Vascular Disorders** | | | | | | | | | | |  |
| Embolism | 47 | 12.83 | (9.63-17.1) | 12.81 | (508.48) | 3.67 | (2) | 12.73 | (10.01) | <0.001 | |
